# Hypoxia-Activated Albumin-Binding Exatecan Prodrug for Cancer Therapy

**DOI:** 10.1021/acsomega.1c05671

**Published:** 2021-12-30

**Authors:** Zhiyang Cheng, Ying Huang, Pingxuan Shao, Lei Wang, Shulei Zhu, Jiahui Yu, Wei Lu

**Affiliations:** Shanghai Engineering Research Center of Molecular Therapeutics and New Drug Development, School of Chemistry and Molecular Engineering, East China Normal University, 3663 North Zhongshan Rd., Shanghai 200062 China

## Abstract

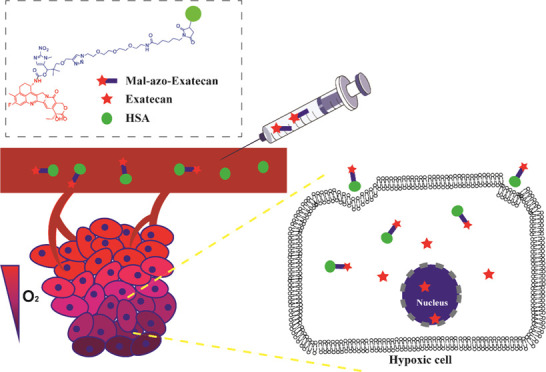

As an effective drug delivery strategy for traditional antitumor drugs, the stimulus-responsive albumin-based prodrugs are getting more and more attention. These prodrugs only release drugs in specific tumor microenvironments, which can prevent premature release of the drug in the circulation. Tumor hypoxia is a fundamental feature of the solid tumor microenvironment. As a hypoxia-activated linker, the 5-position branched linker of 1-methyl-2-nitro-5-hydroxymethylimidazole can be a trigger for albumin-based prodrugs. In this study, we report the synthesis and biological evaluation of the hypoxia-activated albumin-binding prodrug **Mal-azo-Exatecan**. After intravenous administration, the maleimide on the side chain can rapidly bind to endogenous albumin, enabling the prodrugs to accumulate in tumors, where tumor-associated hypoxia microenvironments trigger the selective release of **Exatecan**. The 5-position branched linker of 1-methyl-2-nitro-5-hydroxymethylimidazole as a cleavable linker has high plasma stability and does not cause **Exatecan** release from **HSA-azo-Exatecan** during circulation *in vivo*, avoiding systemic side effects caused by **Exatecan**.

## Introduction

Human serum albumin (HSA) is one of the most important proteins in plasma with multiple functions.^1^ It is also an ideal candidate for drug delivery due to its lack of toxicity and immunogenicity. As a carrier, albumin can provide tumor specificity, reduce drug-related toxicity by altering drug distribution *in vivo* and enhancing cellular uptake, and maintain therapeutic concentrations of therapeutic agents over time.^2^ It also has the potential to extend the half-life of the drug.^3^ Nowadays, drug delivery systems that use albumin as a drug carrier include drug conjugates, drug adducts, albumin-binding derivatives, and nanoparticles.^4−6^ Among them, albumin-binding derivatives especially the albumin-binding prodrugs receive much attention.^7,8^ Albumin-binding prodrugs are designed with the concept of rapid and selective covalent binding to the cysteine 34 position (Cys-34) of serum albumin after intravenous administration to form a macromolecular drug delivery system.^9^ Since covalent binding of a prodrug to albumin greatly reduces the antitumor activity, most of the current research is focused on stimulus-responsive albumin-binding prodrugs.^10,11^ A stimulus-responsive albumin-binding delivery system can effectively accumulate at the tumor site, respond to internal or external stimuli, and release drugs. An advantage of the stimulus-responsive albumin-binding delivery system is that it prevents premature release of the drug in the circulation and avoids systemic toxic effects.^12^

By now, stimulus-responsive albumin-binding prodrugs include bioresponsive, pH-activated, and enzyme-activated albumin-bound prodrugs. For bioresponsive albumin-bound prodrugs, they can be triggered to release the parent drug in a strongly reductive environment in tumor cells.^13^ Xu et al. described bioresponsive albumin-conjugated paclitaxel prodrugs that improved biodistribution and tumor accumulation of paclitaxel and the tumor inhibition rate of this prodrug was approximately 3 times higher than that of the parent drug paclitaxel *in vivo*.^14^ For pH-activated albumin-bound prodrugs, rapid proliferation of cancer cells triggers glycolysis and lowers the pH in the tumor microenvironment, which would facilitate the controlled release of drugs.^15^ Among them, INNO-206, which is an acid cleaved albumin-bound adriamycin prodrug, is currently being assessed in phase III studies for use against sarcoma and gastric cancer.^16^ Enzyme-activated albumin-bound prodrugs rely on the high expression of specific enzymes in tumor tissue, which can be triggered to release the drug in the presence of the corresponding enzymes.^17^ Kratz et al. synthesized a large number of enzymes corresponding to albumin-binding prodrugs including urokinase,^18^ cathepsin B,^19^ and plasmin.^20^ Papot et al. focused on the β-glucuronidase-reactive albumin-binding prodrugs.^21,22^ All in all, the release of stimulus-responsive albumin-binding prodrugs depends on the tumor microenvironment.

Tumor hypoxia is a fundamental feature of the solid tumor microenvironment.^23,24^ The oxygen content in most solid tumors is much lower than that in normal tissues. Treatment of hypoxic tumors becomes one of the most important directions in oncology treatment, including molecular target drugs that act on HIFs and their related signaling pathways and hypoxia-activated prodrugs. At present, the most researched is the hypoxia-activated prodrug, which uses the tumor hypoxic environment to activate the inactive prodrug to release the antitumor drug.^25^ Expression of reductases such as nitroreductase and quinone reductase was much higher in hypoxic tumor tissue than in normal tissue.^26^ In our previous work, we designed a series of hypoxia-sensitive linker chains using 2-nitroimidazole as a framework and found that the 5-position branched linker of 1-methyl-2-nitro-5-hydroxymethylimidazole^27^ was sensitive to the hypoxic environment in solid tumors and had excellent stability in PBS, which has the potential as a trigger for albumin-binding prodrugs.

**Figure 1:**
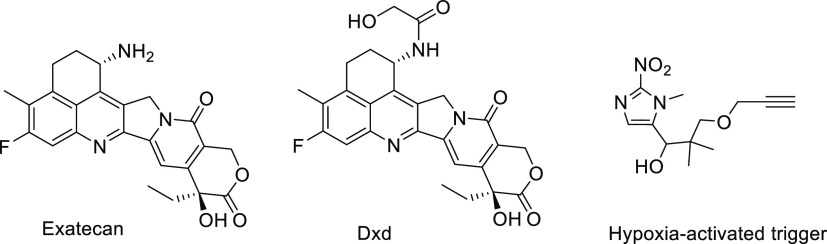
Exatecan, Dxd, and the hypoxia-activated trigger.

Exatecan is a class of camptothecin analogue (Figure 1), which shows excellent antitumor activities in many types of tumors. However, phase II studies and phase III studies do not show ideal antitumor effects due to the ineffective delivery of drugs to tumor tissues.^28^ For the past two years, DS-8201a, an antibody drug conjugate (ADC), using Exatecan derivatives Dxd as effector molecules, has been on the market, resulting in drug delivery systems of Exatecan and its derivatives receiving a lot of attention.^29−31^

**Figure 2:**
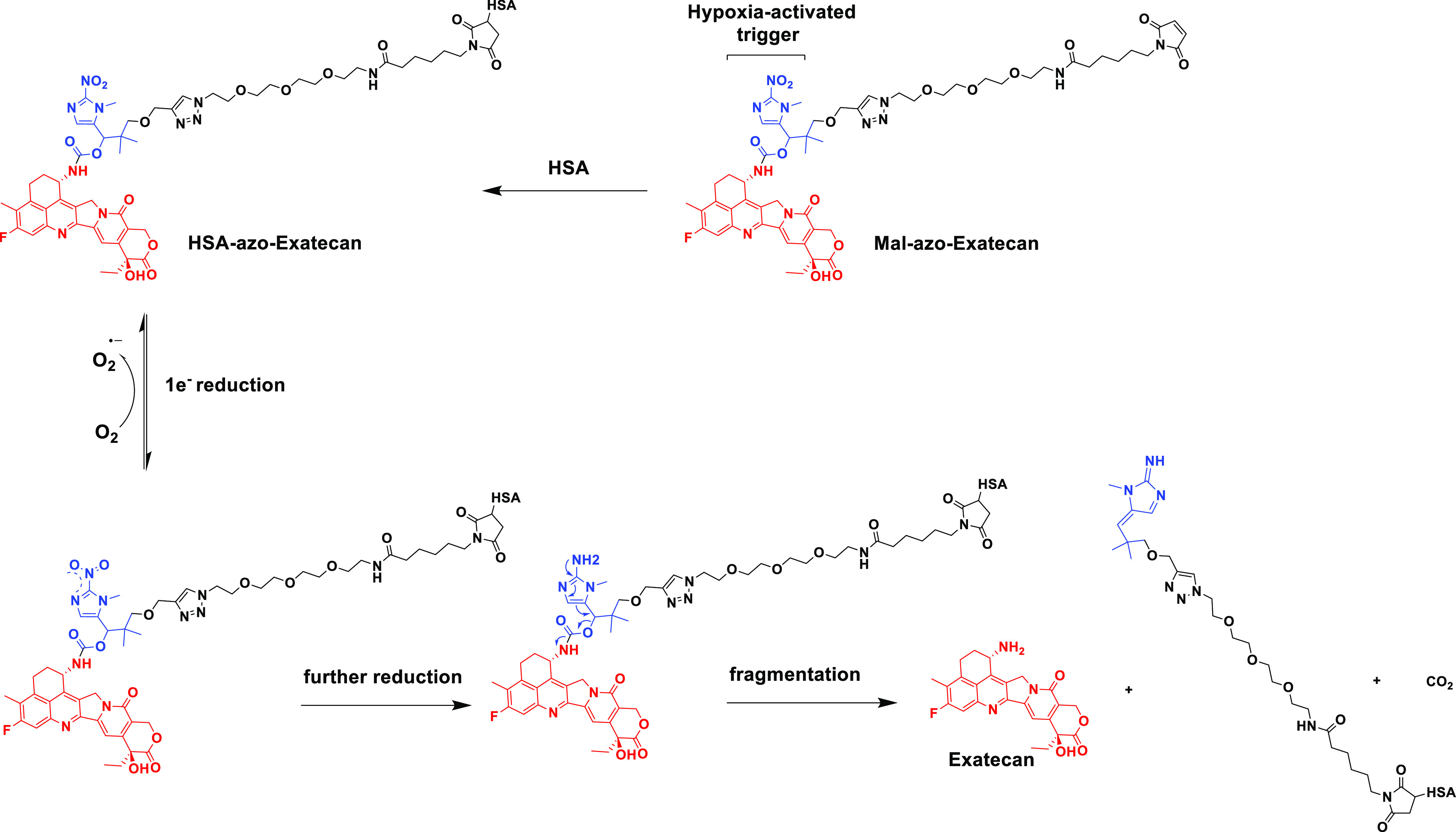
Production of **Mal-azo-Exatecan** and its drug release mechanism.

**Figure 3:**
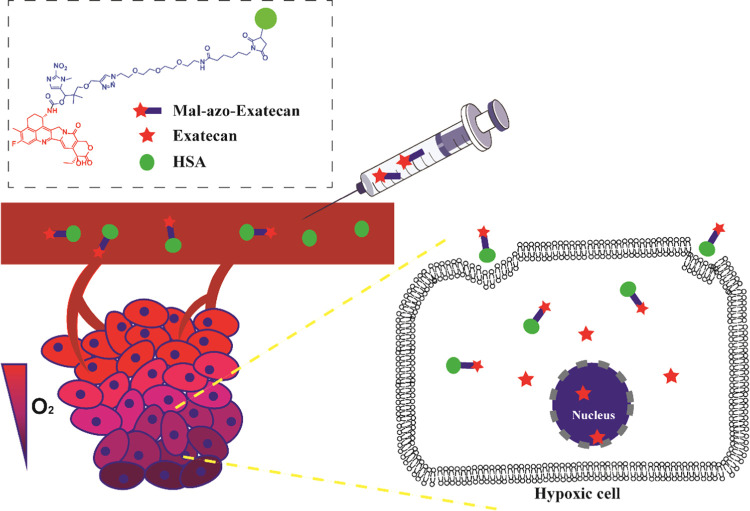
Schematic diagram of the **Mal-azo-Exatecan** therapeutic strategy.

In this study, we reported the synthesis and biological evaluation of a hypoxia-activated albumin-binding prodrug **Mal-azo-Exatecan** (Figure 2). We used the 5-position branched linker of 1-methyl-2-nitro-5-hydroxymethylimidazole as a trigger for hypoxic cleavage, which is bound to the potent camptothecin analogue **Exatecan** using a carbamate bond. The maleimide on the side chain, which could rapidly bind to endogenous albumin after intravenous administration,^32^ formed a large molecule albumin carrier system **HSA-azo-Exatecan** (Figure 3). **HSA-azo-Exatecan** accumulated in tumor tissue through the enhanced permeability and retention (EPR) effect^33^ and the interaction of albumin receptor (gp60),^34^ which could release **Exatecan** triggered by nitroreductase in a hypoxic environment. Meanwhile, we introduced PEG chains to increase the water solubility of the prodrug. The 5-position branched linker of 1-methyl-2-nitro-5-hydroxymethylimidazole as a cleavable linker has high plasma stability and does not cause **Exatecan** release from **HSA-azo-Exatecan** during circulation *in vivo*, avoiding systemic side effects caused by **Exatecan**.

## Results and Discussion

### Binding of **Mal-azo-Exatecan to** Albumin

The rate of binding of **Mal-azo-Exatecan** to the circulating albumin in mouse and human plasma was evaluated *in vitro* using HPLC. As shown in Table 1, 100% of **Mal-azo-Exatecan** was bound to albumin within 2 min postincubation, confirming the fast rate of Michael addition (Table 1 and Figure S1). The binding of **Mal-azo-Exatecan** to HSA was accomplished within 8 min.

**Table 1:** Rate of Binding to Albumin of the Prodrug **Mal-azo-Exatecan** in Murine, Rat, and Human Plasma

	albumin binding	
	time (min)	bound (%)
murine plasma	2	95.81 ± 0.06
	8	99.55 ± 0.11
rat plasma	2	96.77 ± 0.15
	8	99.38 ± 0.12
human plasma	0.25	99.51 ± 0.01
	2	100 ± 0.00
HSA	2	89.41 ± 0.05
	8	99.50 ± 0.13

The molecular weights of **HSA-azo-Exatecan** and HSA were determined by MALDI-TOF MS (Figure S2A). After combining with **Mal-azo-Exatecan** (molecular weight 1140.1934), the maximum strength mass value of HSA changed from 66699.0153 to 67829.8742, and the mass difference was 1130.8589, indicating that a single molecule of **Mal-azo-Exatecan** was bound to a molecule of HSA.

The hydrodynamic diameters and size distribution of **HSA-azo-Exatecan** were measured by dynamic light scattering (DLS). The *Z*-average size of **HSA-azo-Exatecan** was 2.79 nm, and the *Z*-average size of HSA was 2.40 nm (Figure S2B). The ζ-potentials of **HSA-azo-Exatecan** and HSA were similar, which were −11.9 ± 1.7 mV (*n* = 3) and −13.6 ± 0.7 mV (*n* = 3). These data indicate that albumin-bound **Mal-azo-Exatecan** did not change the nano-sized structure of native serum albumin.

### Stability of Albumin Conjugates

The rat plasma albumin conjugate of **Mal-azo-Exatecan** had extremely high stability in rat plasma, and no drug release was detected within 7 days (Table 2 and Figure S3). The conjugate of **Mal-azo-Exatecan** and human plasma albumin also had high stability in human plasma, and no drug release was detected within 7 days. The stability of **Mal-azo-Exatecan** and mouse plasma albumin conjugate was slightly poor in mouse plasma and could release 0.68% **Exatecan** in 7 days. In short, the albumin conjugates formed by the covalent binding of **Mal-azo-Exatecan** with various plasmas showed excellent plasma stability and minimized premature release of payloads.

**Table 2:** Plasma Stability of the Albumin-Drug Conjugates^a^

	% drug released		
	1d	2d	7d
murine plasma	0.04 ± 0.02	0.44 ± 0.01	0.68 ± 0.02
rat plasma	/	/	/
human plasma	/	/	/

a Note: / indicates that no free **Exatecan** was detected.

### *In Vitro* Drug Release

**Figure 4:**
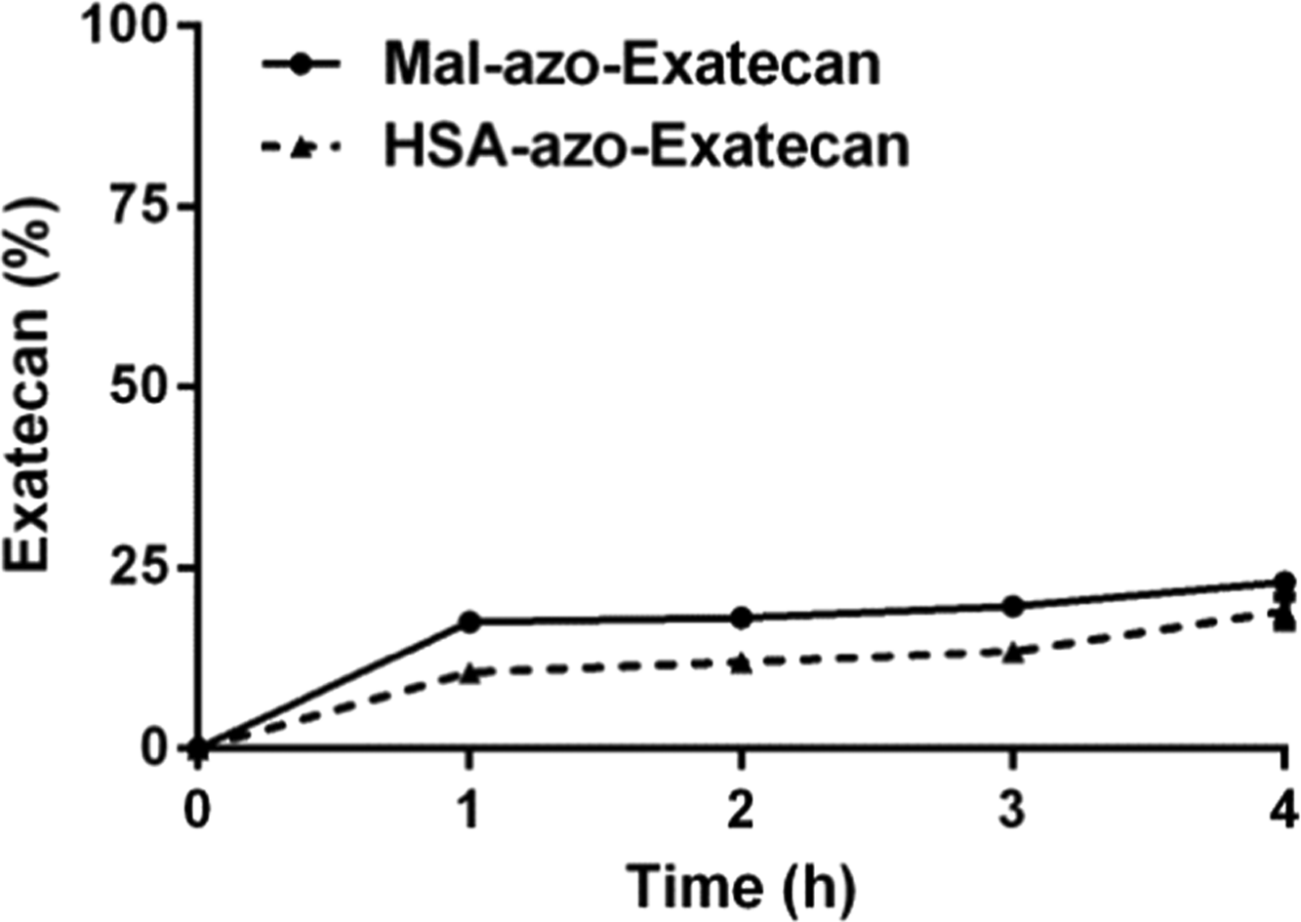
Kinetics of **Exatecan** release from **Mal-azo-Exatecan** and **HSA-azo-Exatecan** in the presence of nitroreductase.

The *in vitro* drug release profiles of **Mal-azo-Exatecan** and **HSA-azo-Exatecan** were investigated under hypoxic conditions (Figure 4). In the presence of nitroreductase, **Mal-azo-Exatecan** released 17.5% of **Exatecan** in 1 h. After 4 h, **Mal-azo-Exatecan** released only 23.1% of **Exatecan**. After binding with HSA, **HSA-azo-Exatecan** released 10.1% of **Exatecan** in 1 h. After 4 h, **HSA-azo-Exatecan** released only 19% of **Exatecan**. In summary, under hypoxic conditions, **HSA-azo-Exatecan** could release **Exatecan** mediated by nitroreductase.

### *In Vitro* Efficacy of **Mal-azo-Exatecan**

We examined the antiproliferative activity of **Mal-azo-Exatecan** against human H460, HT29, A549, HepG2, MCF-7, and Mia PaCa-2 tumor cell lines (Table 3 and Figure S4). Under normal conditions, **Mal-azo-Exatecan** had lower cytotoxic activity than **Exatecan**. Under hypoxic conditions, **Exatecan** had the same cytotoxic activity as normal conditions. However, **Mal-azo-Exatecan** had more potent cytotoxic activity than that under normal conditions, indicating that **Mal-azo-Exatecan** had some hypoxic selectivity. Among them, **Mal-azo-Exatecan** had the highest hypoxia selectivity in NCI-H460 and the HCR (hypoxic cytotoxicity ratio) of 14 times.

**Table 3:** IC_50_ Values of **Exatecan** and **Mal-azo-Exatecan**^a^

	exatecan				Mal-azo-Exatecan	
cell lines	IC_50_(air) (nM)	IC_50_(N_2_) (nM)	HCR	IC_50_(air) (μM)	IC_50_(N_2_) (μM)	HCR
H460	1.47 ± 0.40	1.18 ± 0.10	1.25	49.86 ± 12.91	3.45 ± 0.32	14.43
HT29	13.24 ± 2.00	18.31 ± 6.32	0.72	95.42 ± 4.87	51.33 ± 6.65	1.86
A549	10.82 ± 0.90	11.93 ± 0.77	0.91	>100	17.22 ± 1.59	>5.88
HepG2	96.48 ± 7.02	154.86 ± 25.21	0.62	>100	32.52 ± 7.84	>3.08
MCF-7	38.34 ± 5.88	87.04 ± 4.83	0.44	>100	71.97 ± 9.33	>1.39
Mla PaCa-2	0.72 ± 0.23	0.25 ± 0.05	2.88	13.38 ± 2.10	4.99 ± 1.14	2.68

a Note: HCR, hypoxic cytotoxicity ratio; HCR= IC_50_ (Air)/IC_50_ (N_2_).

### *In Vivo* Drug Accumulation and Biodistribution Study

The *in vivo* distribution properties and tumor selectivity of **Exatecan** and **Mal-azo-Exatecan** were evaluated in a mouse model bearing H460-transplanted tumors (Figure S5). Free **Exatecan** was mainly in the livers and spleens, and the content of **Exatecan** in tumors was low. In contrast, **Mal-azo-Exatecan** showed a higher tumor accumulation than free **Exatecan** in tumors. At 2, 6, 24, and 48 h, the cumulative amount of **Exatecan** in the tumor tissue of the **Mal-azo-Exatecan** group was 6-, 21-, 46-, and 72-fold higher than that of the **Exatecan** group, respectively. These results suggested that this prodrug enhances the selectivity *in vivo* and improves the preferential accumulation of the prodrug in tumors.

**Figure 5:**
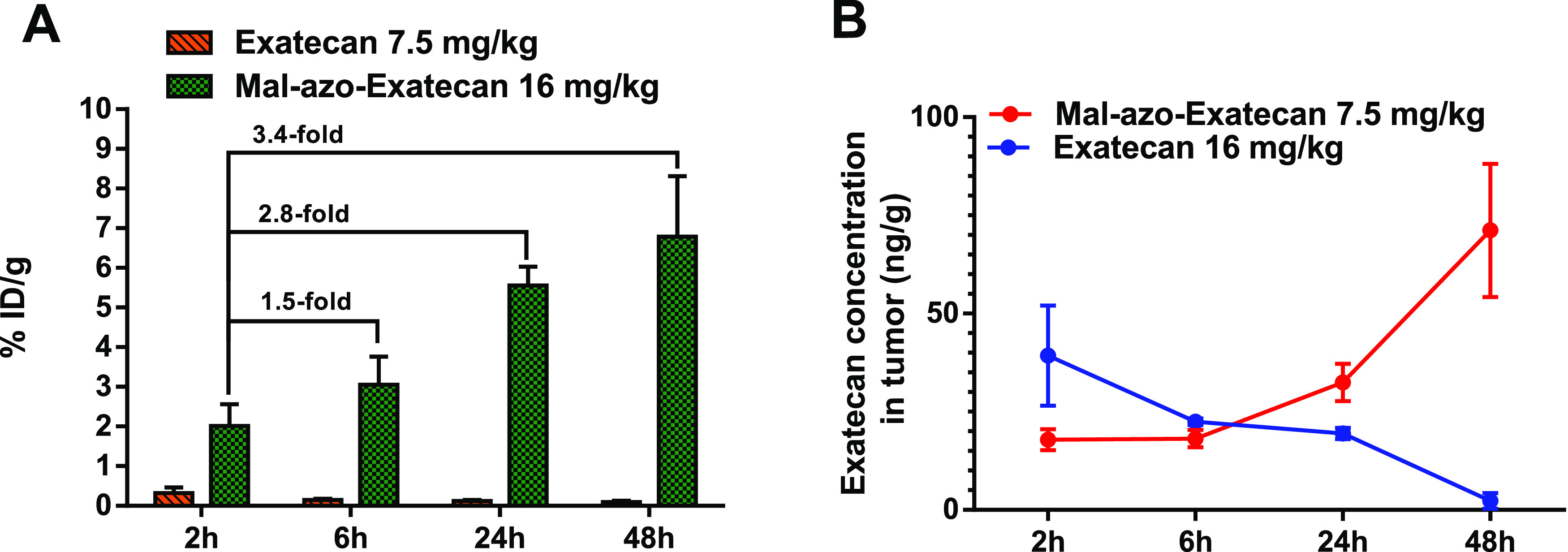
(A) Tumour accumulation by **Exatecan** (7.5 mg/kg) and **Mal-azo-Exatecan** (16 mg/kg) in the H460 transplant tumor model. (B) Release of **Exatecan** in tumors.

Meanwhile, the cumulative amount of **Mal-azo-Exatecan** increased over time. At 6, 24, and 48 h, the cumulative amount of **Mal-azo-Exatecan** in the tumor tissue of the **Mal-azo-Exatecan** group was 1.5-, 2.8-, 3.4-fold higher than that of the cumulative amount of **Mal-azo-Exatecan** at 2 h (Figure 5). Unfortunately, the release of the prodrug in the tumor is not ideal. At 2 and 6 h, the release of **Exatecan** in the tumor tissue of the **Mal-azo-Exatecan** group was lower than the cumulative amount of the **Exatecan** group. As time went on, the content of **Exatecan** of the **Exatecan** group gradually reduced. However, at 24 and 48 h, the release of **Exatecan** in the tumor tissue of the **Mal-azo-Exatecan** group increased. These results suggested that **Mal-azo-Exatecan** could improve the preferential accumulation of the prodrug in tumors, but the rate of drug release was low.

### Inhibition effect of **Mal-azo-Exatecan**

**Figure 6:**
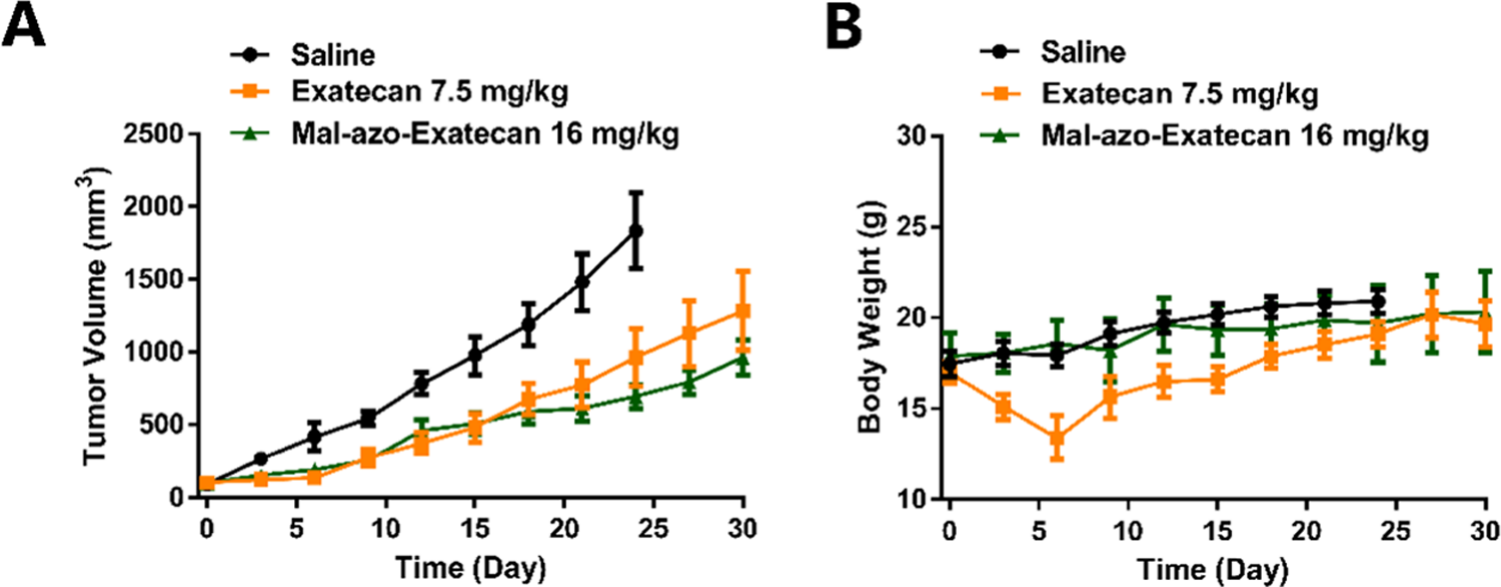
(A) Tumor volume of H460-transplanted tumor-bearing BALB/C nude mice of the three groups over 18 days. (B) Body weight of the H460-transplanted tumor-bearing BALB/C nude mice of the three groups over 18 days.

Due to the highest hypoxic selectivity of **Mal-azo-Exatecan** in the H460 cell line, the *in vivo* antitumor activity of **Mal-azo-Exatecan** was assessed in H460-transplanted tumor-bearing BALB/C nude mice. Mice in the saline, **Exatecan**, and **Mal-azo-Exatecan** groups were given tail vein injections every three days. Mice in the saline and **Mal-azo-Exatecan** groups were given five consecutive doses and then stopped for a fortnight. Mice in the **Exatecan** group lost more than 20% of their body weight before the third dose and were stopped once. Mice in the **Exatecan** group were stopped for a fortnight after a total of four doses. The tumor volume of the saline group grew rapidly over time (Figure 6A). However, the antitumor effects of the **Mal-azo-Exatecan** group were similar to those of the **Exatecan** group. On day 30, the tumor volume of the **Mal-azo-Exatecan** group was 961 ± 297 mm^3^ and the tumor volume of the **Exatecan** group was 1281 ± 666 mm^3^. The weight of the mice in the **Exatecan** group decreased significantly and gradually recovered after stopping the drug once. Meanwhile, the body weight of the mice in the **Mal-azo-Exatecan** group increased relatively steadily (Figure 6B), indicating that **Mal-azo-Exatecan** was less toxic than **Exatecan**.

### Histological Analysis

**Figure 7:**
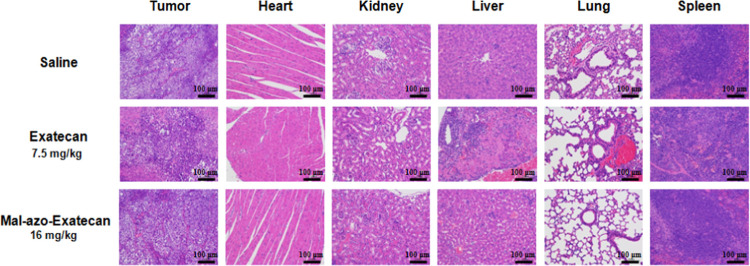
H&E staining of the main organs of H460-transplanted tumor-bearing BALB/C nude mice from each group after treatment.

Potential toxicities were evaluated by histological evaluation of hematoxylin and eosin (H&E)-stained tissues (Figure 7). The H&E staining images of major organs indicated that the **Exatecan** group exhibited massive hepatocyte necrosis with nuclei fragmentation and lysis and no clear pathologic changes were detected in the **Mal-azo-Exatecan** group. These results suggest that **Mal-azo-Exatecan** has low pathological toxicity.

## Conclusions

In this study, we developed a new hypoxia-activated albumin-binding prodrug **Mal-azo-Exatecan** leading to the *in vivo* formation of the corresponding albumin conjugate and the release of the camptothecin derivative **Exatecan** in the tumor tissue. In hypoxic conditions, **Mal-azo-Exatecan** showed more potent antitumor activities than the normal conditions, which indicated that the prodrug had some hypoxic selectivity. In the antitumor effect evaluation in H460-transplanted tumor-bearing BALB/C nude mice, **Mal-azo-Exatecan** had lower toxicity and better antitumor effect than **Exatecan**, but it did not successfully inhibit tumor growth. The *in vivo* distribution experiment showed that the prodrug did not release **Exatecan** well in the tumor, leading to its poor antitumor effect *in vivo*. This phenomenon might be due to the fact that the level of hypoxia in this tumor model was not very high, resulting in a slow release of the prodrug after accumulation, which was not sufficient to inhibit tumor growth. Therefore, more consideration needs to be given to whether the tumour hypoxia microenvironment can release the drug rapidly in subsequent design.

## Experimental Section

### Materials

Methanol (MeOH), ethanol (EtOH), ethyl acetate (EA), anhydrous sodium sulfate (Na_2_SO_4_), dichloromethane (DCM), hydrochloric acid (HCl), tetrakis (acetonitrile) copper(I) hexafluoroho phosphate (Cu(MeCN)_4_PF_6_), petroleum ether (PE, 60–90), sodium bicarbonate (NaHCO_3_), and *N*,*N*-diisopropylethylamine (DIPEA) were obtained from Sinopharm Chemical Reagent Co., Ltd. (Shanghai, China). 4-Dimethylaminopyridine (DMAP) and *N*-succinimidyl 6-maleimidohexanoate (EMCS) were obtained from Suzhou Highfine Biological Co., Ltd. (Suzhou, China). *N*,*N*-Dimethylacetamide (DMF), deuterated chloroform (CDCl_3_, 99.8%), deuterium dimethyl sulfoxide (DMSO-*d*_6_, 99.8%), β-glucuronidase from *Escherichia coli* (5000 units), 3-(4,5-dimethylthiazol-2-yl)-2,5-diphenyltetrazolium bromide (MTT), and albumin from human serum were purchased from Sigma-Aldrich (St. Louis, MO). Acetonitrile (ACN, HPLC grade) and trifluoroacetic acid (TFA, HPLC grade) were purchased from J&K Scientific Ltd. (Beijing, China). Culture media, penicillin–streptomycin, and 4% paraformaldehyde solution were obtained from HyClone (Logan, Utah). Foetal bovine serum (FBS) and trypsin were obtained from Gibco (BRL, MD). An Amicon Ultra-4 centrifugal unit (MWCO 10 kDa) was obtained from Merck Chemicals Co., Ltd. (Shanghai, China).

### Synthesis of **Mal-azo-Exatecan**

**Scheme 1:**
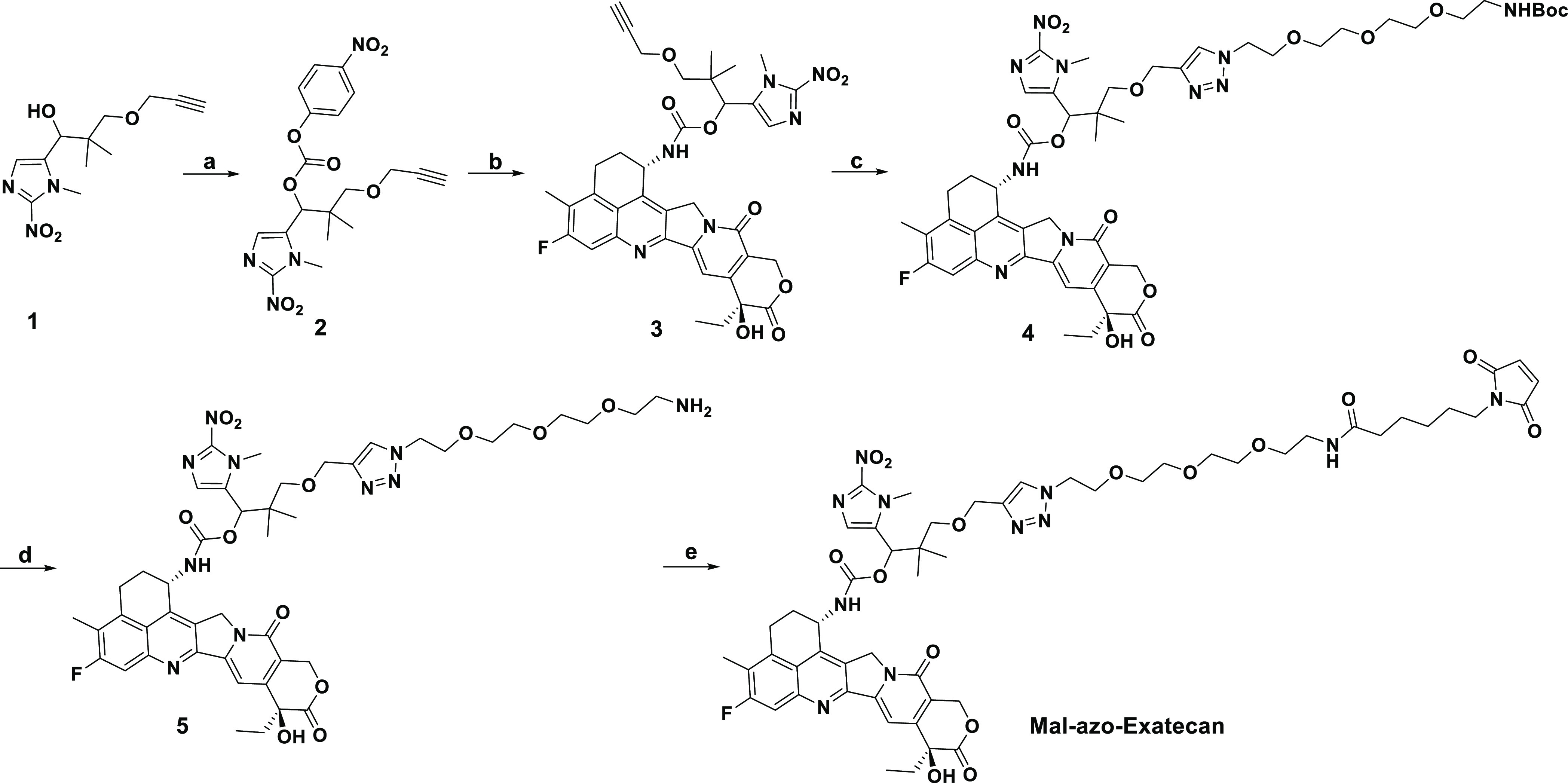
Reagents and Conditions: (a) 4-Nitrophenyl Chloroformate, Pyridine, DCM, rt; (b) **Exatecan**, DMAP, DMF, rt; (c) N_3_PEG_4_NHBoc, Cu(MeCN)_4_PF_6_, DCM, rt; (d) TFA, DCM, rt; and (e) EMCS, DIPEA, DCM, rt

The detailed synthesis of the **Mal-azo-Exatecan** is illustrated in Scheme 1. The starting material compound **1** prepared according to previous literature was reacted with 4-nitrophenyl chloroformate in the presence of pyridine to yield compound **2**. Compound **2** was reacted with **Exatecan** in the presence of DMAP in DMF to yield compound **3**. Compound **3** was reacted with N_3_-PEG_4_-NH-BOC to form compound **4** by a click reaction. After complete deprotection using TFA and reaction with EMCS, **Mal-azo-Exatecan** was obtained. More details can be found in the Supporting Information.

### Cell Lines and Culture

A549 (human lung cancer cell line) and H460 (human large cell lung cancer cell line) cells were cultured in RPMI-1640 with 10% FBS and 1% Pen-Strep. The human colon cancer cell line HT29 was cultured in McCoy’s 5A with 10% FBS and 1% Pen-Strep. The human breast cancer cell line MCF-7 was cultured in MEM with 10% FBS and 1% Pen-Strep. The human hepatocarcinoma cell line HepG2 and human pancreatic cancer cell line were cultured in DMEM with 10% FBS and 1% Pen-Strep. All of the cell lines were obtained from the Chinese Academy of Science Cell Bank for Type Culture Collection (Shanghai, China) and maintained in a humidified atmosphere at 37 °C and 5% CO_2_.

### HPLC Determination of Nitroreductase-Dependent Drug Release

Nitroreductase (50 U, purchased from Sigma-Aldrich) and NADPH (1 mM, purchased from Sigma-Aldrich) were added to a solution of **Mal-azo-Exatecan** or **HSA-azo-Exatecan** (100 μM in PBS, pH 7.4) and incubated in the three-gas incubator (1% O_2_) at 37 °C. The samples (100 μL) were taken at a specific point, immediately added to 100 μL of cold methanol, vortexed for 1 min, and then centrifuged for 30 min at 4 °C. The release of **Exatecan** over time was detected by HPLC (Method 1).

### *In Vitro* MTT Cytotoxicity Assay

HT460, HT29, A549, MCF-7, Mia PaCa-2 (4 × 10^3^ cells/well), and HepG2 (7 × 10^3^ cells/well) cells were seeded in a 96-well plate. Twenty-four hours later, cells were exposed to **Exatecan** or **Mal-azo-Exatecan** and incubated in a normal and anaerobic incubator, which was further incubated for 72 h. Cell viability and proliferation behavior were assessed by the MTT assay. The absorbance was measured at 570 nm using an automated microplate reader (Spectra Max M5, Molecular Devices). The cell viability was calculated using eq 1 as follows
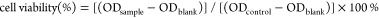
1

The OD_sample_ is the optical density (OD) value of cells treated with various formulations, the OD_control_ is the OD value of cells incubated with culture media, and the OD_blank_ is the OD value of the culture media alone.

### *In Vivo* Antitumor Efficacy

When the tumor sizes reached ∼100  mm^3^, the H460 tumor-bearing mice were randomly assigned into control and treatment groups, with six mice per group. Control groups were given vehicle alone, and treatment groups received the indicated compounds (iv). Three groups were designed and received intravenous injections of 0.9% physiological saline solution (control group), 7.5 mg/kg **Exatecan** hydrochloride, or 16 mg/kg **Mal-azo-Exatecan** twice per week for 2 weeks (five doses in total). The length and width of the tumor volume were determined using Verniers calipers twice a week. The tumor volume (*V*) was calculated using eq 2 as follows
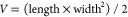
2

The body weight was recorded twice a week. At the experimental endpoint, all of the mice were sacrificed and tumors and major tissues were excised for weight measurement and further examination. The tumor growth inhibition rate (IR) was calculated based on the weight of the tumor on the last day.

### Histological Analysis

The main organs (heart, liver, spleen, lung, and kidney) and tumors were excised, fixed in 4% formaldehyde, embedded in paraffin, and sectioned into slices at a thickness of 3 μm for further hematoxylin and eosin (H&E) staining. The H&E-stained samples were observed under an optical microscope.

## Supporting Information Available

Methods: general methods for synthesis and HPLC methods; *In vitro* data: binding kinetics of Mal-azo-Exatecan to albumin in murine, rat, and human plasma, stability of albumin conjugates in murine, rat, and human plasma, characterization of HSA-azo-Exatecan, albumin binding study using HPLC analysis, stability of albumin conjugates in murine, rat, and human plasma, IC_50_ data; *In vivo* data: xenografts model, *in vivo* biodistribution study, *in vivo* distribution properties and tumor selectivity, and statistical analysis; and experimental: synthesis of Mal-azo-Exatecan, NMR raw spectra, and high-resolution mass spectra of Mal-azo-Exatecan (PDF)
