# Supplementary material for: Hypoxia-Activated Albumin-Binding Exatecan Prodrug for Cancer Therapy
Source: ACS Omega. 2021 Dec 30;7(1):1082–9. doi: 10.1021/acsomega.1c05671 (PMC8757358; doi:10.1021/acsomega.1c05671)
Supplement: Supplementary file 1 — ao1c05671_si_001.pdf [file ao1c05671_si_001.pdf]

# A hypoxia-activated albumin-binding exatecan prodrug for cancer therapy

Zhiyang Cheng [1,†], Ying Huang [1,†], Pingxuan Shao [1], Lei Wang [1,*], Shulei Zhu [1], Jiahui Yu [1], Wei Lu [1,*]

[1] *Shanghai Engineering Research Center of Molecular Therapeutics and New Drug Development, School of Chemistry and Molecular Engineering, East China Normal University, 3663 North Zhongshan Rd., Shanghai, China, 200062*

* Corresponding authors:

E-mail: wlu@chem.ecnu.edu.cn;

E-mail: lwang@chem.ecnu.edu.cn

†These authors contributed equally to this work.

## Supporting information

## 1. Methods

### 1.1. General methods for synthesis

$^1$H and $^{13}$C nuclear magnetic resonance (NMR) spectra were recorded on a Bruker DRX-400 MHz spectrometer (400 and 101 MHz, respectively) using $CDCl_3$, $CD_3OD$ or DMSO-$d_6$ as solvents with TMS as an internal standard. Chemical shifts were reported as d (ppm) and spin-spin coupling constants as $J$ (Hz) values. The mass spectra (MS) were recorded on a Waters SDQ mass spectrometer and high resolution mass spectra (HRMS) were recorded on Waters SYNAPT G2 ESI-TOF-MS analyzer. Melting points were taken on a SGW X-4 melting point apparatus, uncorrected and reported in degrees Centigrade. Column chromatography was performed with silica gel (200–300 mesh). All the starting materials are commercially available and were used without further purification.

### 1.2. HPLC Methods

**HPLC (Method 1)**: Agilent ZORBAX SB-C18 column (250 × 4.6 mm, 5 μm); mobile phase: 0.1% TFA in water (phase A) and acetonitrile (phase B); elution gradient of phase B: 0–2.0 min: 5%, 2.0–12.0 min: 5–95%, 12.0–15.0 min: 95%, 15.0–15.1 min: 5%, 15.1–22.0 min: 5%, 42.0 min: method end. Flow rate=1.0 mL/min. Column oven: 35 °C.

**HPLC (Method 2)**: Agilent ZORBAX SB-C18 column (250 × 4.6 mm, 5 μm); mobile phase: 0.1% TFA in water (phase A) and acetonitrile (phase B); elution gradient of phase B: 0–2.0 min: 5%, 2.0–32.0 min: 5–95%, 32.0–35.0 min: 95%, 35.0–35.1 min: 5%, 35.1–42.0 min: 5%, 42.0 min: method end. Flow rate=1.0 mL/min. Column oven: 35 °C.

**HPLC (Method 3)**: Agilent ZORBAX SB-C18 column (250 × 9.4 mm, 5 μm); mobile phase: 0.1% TFA in water (phase A) and acetonitrile (phase B); elution gradient of phase B: 0–2.0 min: 5%, 2.0–32.0 min: 5–95%, 32.0–35.0 min: 95%, 35.0–35.1 min: 5%, 35.1–42.0 min: 5%, 42.0 min: method end. Flow rate=1.0 mL/min. Column oven: 35 °C.

## 2. *In vitro* data

### 2.1. Binding kinetics of Mal-azo-Exatecan to albumin in murine, rat and human plasma

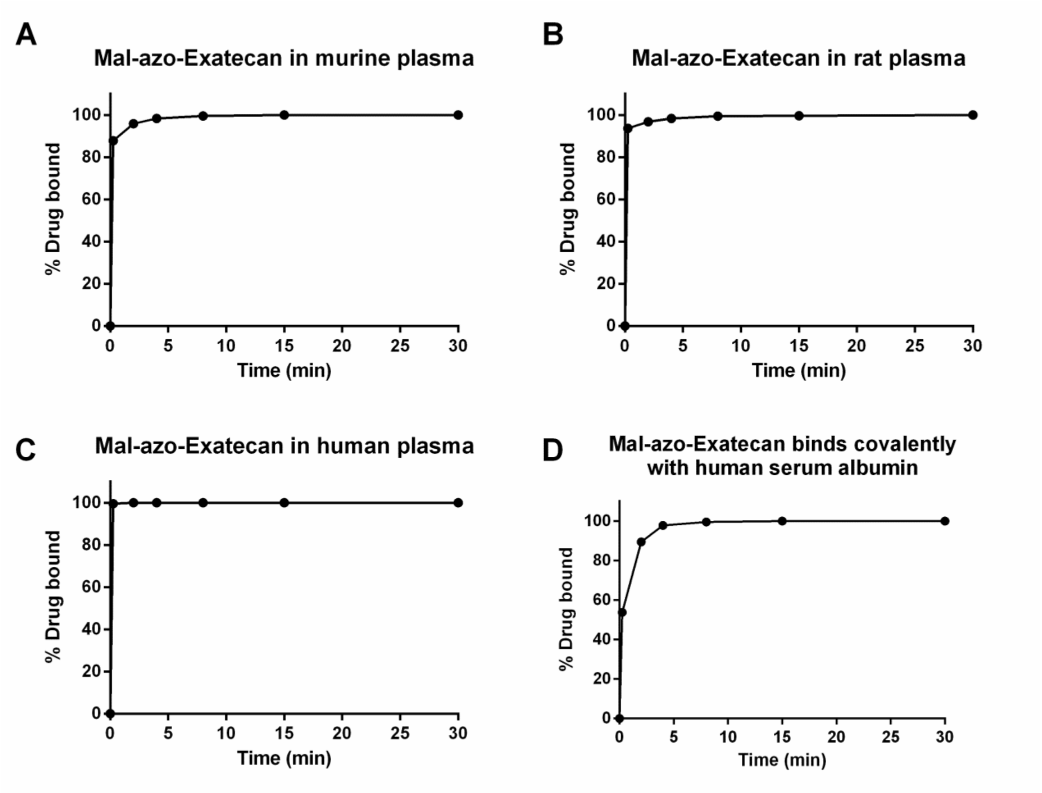


**Figure S1**. (A) Binding kinetic profiles of **Mal-azo-Exatecan** in murine plasma; (B) Binding kinetic profiles of **Mal-azo-Exatecan** in rat plasma; (C) Binding kinetic profiles of **Mal-azo-Exatecan** in human plasma; (D) Binding kinetic profiles of **Mal-azo-Exatecan** in 5% human serum albumin.

## 2.2. Stability of albumin conjugates in murine, rat and human plasma

After the plasma was preheated at 37°C for 30 minutes, 40 μL of **Mal-azo-Exatecan** stock solution (1 mM) was added to 360 μL of each plasma and allowed to react for 30 min at 37°C. Samples (40 μL) were taken at chosen time points, snap-frozen with liquid nitrogen and then stored at -20°C until the end of the experiment. At the end of the experiment, samples were removed from the freezer and allowed to thaw at room temperature. The samples were immediately added to 160 μL of cold acetonitrile, vortexed for 1 min, and then centrifuged for half an hour at 4°C. The release of **Exatecan** was detected by HPLC (Method 1).

## 2.3. Characterization of HSA-azo-Exatecan

**HSA-azo-Exatecan** was prepared by incubating **Mal-azo-Exatecan** (100 μM) with HSA (70 μM) in PBS (pH 7.4) for 1 h. The unreacted **Mal-azo-Exatecan** and salts were removed by centrifugation (20 min, 3000 rpm) in an Amicon Ultra-4 centrifugal unit (MWCO 10 kDa, EMD Millipore, Billerica, MA). The solution in the insert was collected and lyophilized to obtain **HSA-azo-Exatecan** as a yellow powder. The masses of **HSA-azo-Exatecan** and HSA were analysed by MALDI-TOF MS (AB Sciex 4800 MALDI TOF/TOF™) and reconstituted in 1 mL PBS (pH 7.4) for HPLC analysis (Method 2).

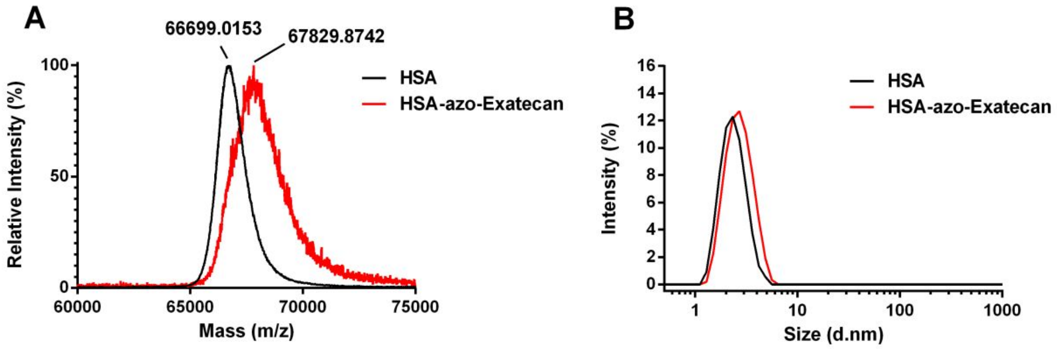


**Figure S2.** (A) MALDI-TOF MS spectra of HSA (black) and **HSA-azo-Exatecan** (red); (B) Hydrodynamic sizes of HSA (black) and **HSA-azo-Exatecan** (red).

## 2.4. Albumin binding study using HPLC analysis

Human, murine and rat plasma was obtained from healthy volunteers, female BALB/c mice and male Sprague-Dawley rats. A total of 360 μL of the plasma or 5%

HSA solution was incubated at 37°C using an Eppendorf Thermomixer C. After 30 min of incubation, 40 μL of the **Mal-azo-Exatecan** solution (1 mM in PBS, pH 7.4) was added to the plasma. Samples (40 μL) were taken after 15 s, 2 min, 4 min, 8 min, 15 min and 30 min (total of six samples). The samples were immediately added to 160 μL cold acetonitrile, vortexed for 1 min, and then centrifuged for half an hour at 4°C. The amount of prodrug remaining in the supernatant was detected by HPLC (Method 1).

## 2.5. Stability of albumin conjugates in murine, rat and human plasma

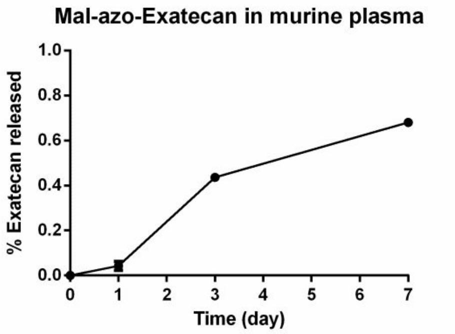


**Figure S3**. Stability profiles of albumin conjugates in murine, rat and human plasma.

## 2.6. $IC_{50}$ data

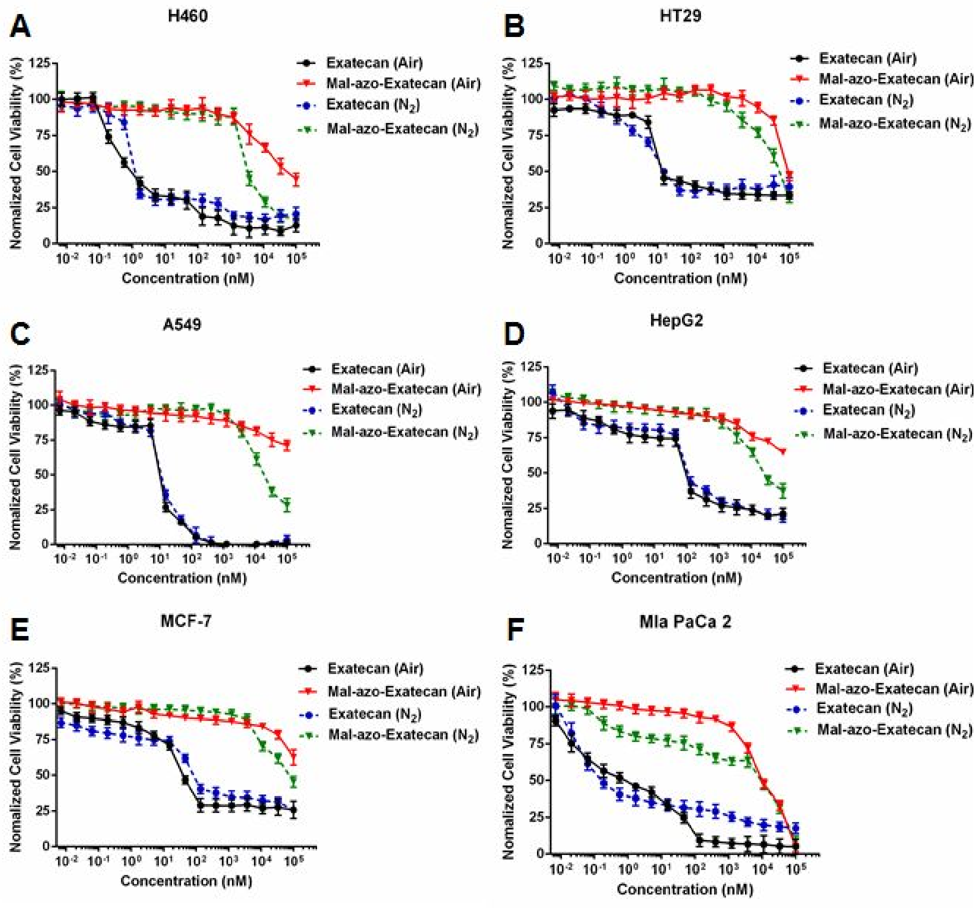

**Figure S4.** Anti-proliferative activity of **Exatecan** and **Mal-azo-Exatecan** under air and $N_2$ condition against (A) human H460 tumour cell lines; (B) human HT29 tumour cell lines; (C) human A549 tumour cell lines; (D) human HepG2 tumour cell lines; (E) human MCF-7 tumour cell lines; (F) human Mia PaCa-2 tumour cell lines.

# 3. *In vivo* data

## 3.1. Xenografts model

Female BALB/C mice (~ 6 weeks, 20 ± 2 g) were purchased from SLAC Laboratory Animal Co., Ltd. and fed in the Experimental Animal Center of East China Normal University under specific pathogen-free conditions. Animal experimental procedures were approved by the Institutional Animal Care and Use Committee of East China Normal University. NCl-H460 cells were inoculated subcutaneously into the right armpit of mice at an inoculation quantity of $5 \times 10^6$ cells per mouse.

## 3.2. *In vivo* biodistribution study

To assess the accumulation and biodistribution of **Exatecan** and **Mal-azo-Exatecan** *in vivo*, studies were performed in subcutaneous H460-inoculated female BALB/C mice. After the tumour volume was approximately 200 $mm^3$, mice were randomly assigned to two groups (n=12): (1) 7.5 mg/kg **Exatecan** methanesulfonate and (2) 16 mg/kg **Mal-azo-Exatecan**. After intravenous injection via lateral tail veins, the tumour-bearing mice were sacrificed at specific times, and the heart, liver, spleen, lung, kidney and tumour tissues were excised, washed with phosphate buffered saline (pH 7.4), weighed after drying and homogenized in a 0.5 mL mixture of DMSO and $H_2O$ (v/v, 1:1). After centrifugation of the mixture, the supernatant was analysed using a fluorescence detector (excitation: 360 nm, emission: 450 nm), and the drug content was quantified according to the respective standard calibration curve. The percent injected dose (%ID) and the percent ID per gram (%ID/g) were calculated using the following eq. 1. and eq. 2.:

$$\%ID = (\text{dose in tissue sample/injected dose}) \times 100 \quad (1)$$

$$\%ID/g = \%ID/\text{weight of tissue (g)} \quad (2)$$

## 3.3. *In vivo* distribution properties and tumour selectivity

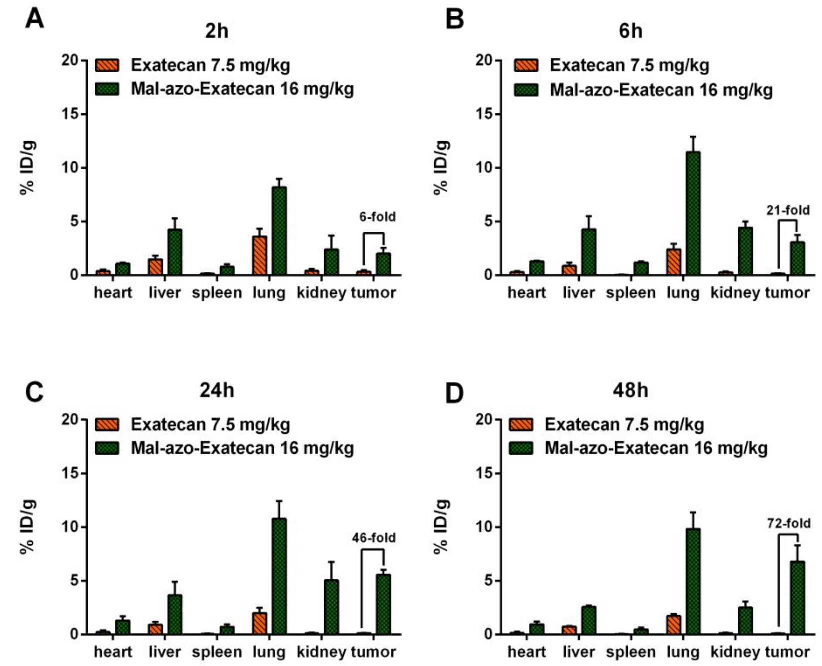


**Figure S5.** Drug concentration in tumour tissues and major organs after i.v. injection of **Exatecan** methylsulfonate and **Mal-azo-Exatecan** at (A) 2h; (B) 6h; (C) 24h; (D) 48h.

### 3.4. Statistical analysis

All experiments were conducted three times, and data are presented as the means ± SD. GraphPad Prism version 6.0e (GraphPad Software, San Diego, CA) was used for statistical calculations using Student's *t*-test and ANOVA. The differences were considered significant when *p < 0.05, **p < 0.01, ***p < 0.001, or ****p < 0.0001.

## 4. Experimental

### 4.1. Synthesis of Mal-azo-Exatecan

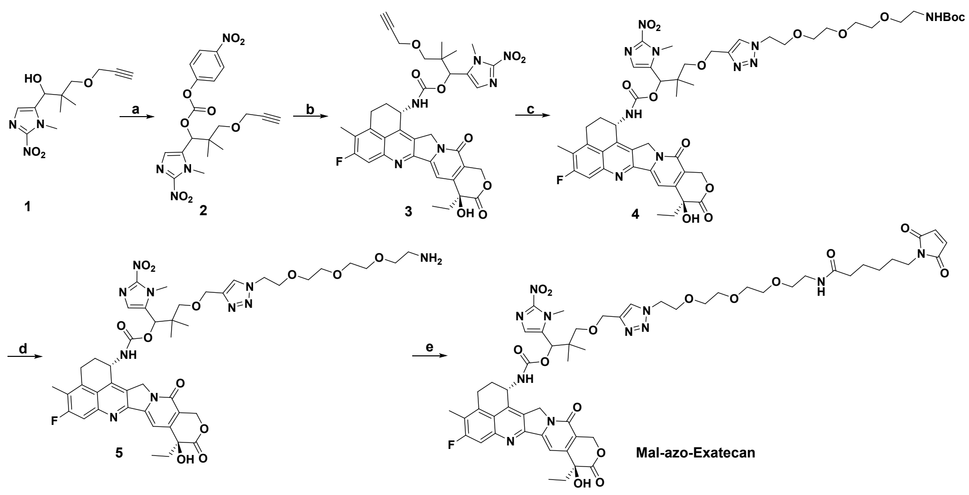

**Scheme S1**. Reagents and conditions: a) 4-nitrophenyl chloroformate, pyridine, DCM, rt; b) **Exatecan**, DMAP, DMF, rt; c) $N_3PEG_4NHBoc$, $Cu(MeCN)_4PF_6$, DCM, rt; d) TFA, DCM, rt; e) EMCS, DIPEA, DCM, rt.

**Compound 2 -** Compound **1** (534 mg, 2 mmol) and 4-nitrophenyl chloroformate (804 mg, 4 mmol) were added to a solution of pyridine (330 μL, 4 mmol) in DCM (40 mL). The mixture was stirred at room temperature for 12 hours and evaporated under reduced pressure to give the crude product. The crude product was purified by column chromatography in 10:1 PE/EA (v/v) to give 622 mg compound **2**, which was used in the subsequent reaction without further purification. MS (ESI): *m/z* 433.4 ($M^+$ +1).

**Compound 3 -** Compound **2** (432 mg, 1.0 mmol) was dissolved in dry DMF (15 mL) under nitrogen atmosphere, **Exatecan** (531 mg, 1.0 mmol) and DMAP (183 mg, 1.5 mmol) were added. The mixture was stirred at room temperature for 3 hours. The solvent was added with $H_2O$ (75 mL), extracted with EA (4 × 30 mL), dried over $Na_2SO_4$, filtered, and the solvent was evaporated under reduced pressure. The crude material was purified by column chromatography in 50:1 DCM/MeOH (v/v) to give **3** (677 mg, yield 90%) as a light yellow solid. $^1$H NMR (400 MHz, DMSO) δ 7.93 (d, J = 7.4 Hz, 1H), 7.75 (d, J = 10.9 Hz, 1H), 7.29 (s, 1H), 7.18 – 7.07 (m, 1H), 6.51 (s, 1H), 5.40 (s, 2H), 5.34 – 5.11 (m, 3H), 4.63 (d, J = 2.9 Hz, 1H), 4.21 – 4.13 (m, 1H), 4.10 – 3.82 (m, 6H), 3.43 (t, J = 2.1 Hz, 1H), 3.27 – 3.06 (m, 2H), 2.37 (s, 3H), 2.27 – 2.06 (m, 2H), 1.97 – 1.74 (m, 2H), 1.02 (s, 3H), 0.94 – 0.81 (m, 6H). $^{13}$C NMR (101 MHz, DMSO) δ 172.39, 162.75, 160.27, 156.57, 155.94, 152.34, 149.84, 147.86, 145.94, 144.95, 140.63, 136.17, 135.64, 128.20, 123.71, 123.52, 119.09, 109.87, 96.63, 79.34, 77.78, 75.09, 72.29, 65.21, 56.62, 49.57, 47.16, 34.89, 30.32, 28.23, 23.67, 20.56, 20.38, 19.92, 19.71, 10.85, 7.70. MS (ESI): *m/z* 729.5 ($M^+$ +1).

**Compound 4 -** Compound **3** (728 mg, 1.0 mmol) was dissolved in dry DCM (50 mL) under nitrogen atmosphere, $N_3PEG_4NHBoc$ (636 mg, 2.0 mmol) and $Cu(MeCN)_4PF_6$ (558 mg, 1.5 mmol) were added. The mixture was stirred at room temperature for 12 hours. The solvent was evaporated under reduced pressure. The crude material was purified by column chromatography in 50:1 DCM/MeOH (v/v) to give **4** (732 mg, yield 70%) as a light yellow solid. $^1$H NMR (400 MHz, CDCl3) δ 7.70 (d, J = 21.4 Hz, 1H), 7.52 – 7.33 (m, 2H), 7.11 (d, J = 11.5 Hz, 1H), 6.39 (d, J = 28.5 Hz, 1H), 5.58 – 5.49 (m, 1H), 5.28 – 5.16 (m, 2H), 5.07 – 4.86 (m, 3H), 4.70 – 4.37 (m, 6H), 4.25 – 3.94 (m, 5H), 3.81 – 3.70 (m, 2H), 3.60 – 3.53 (m, 8H), 3.51 – 3.45 (m,

2H), 3.28 – 3.06 (m, 4H), 2.34 (s, 3H), 2.20 (s, 2H), 1.98 – 1.87 (m, 2H), 1.38 (s, 9H), 1.08 – 0.99 (m, 6H), 0.90 (d, J = 7.3 Hz, 3H). $^{13}$C NMR (101 MHz, CDCl3) δ 173.55, 163.54, 163.51, 161.04, 161.01, 157.04, 156.97, 156.07, 155.98, 152.14, 150.09, 150.02, 148.41, 148.28, 145.86, 145.81, 145.47, 145.38, 143.70, 143.50, 139.62, 139.28, 136.60, 136.47, 135.17, 129.21, 129.16, 125.18, 124.99, 124.81, 124.57, 124.52, 121.22, 121.13, 119.55, 119.38, 110.13, 109.99, 109.89, 109.75, 97.84, 79.27, 75.76, 75.41, 72.83, 72.80, 70.69, 70.42, 70.40, 70.17, 69.98, 69.23, 69.21, 66.03, 65.85, 62.57, 50.22, 50.17, 49.48, 47.86, 40.48, 40.26, 35.28, 35.20, 31.40, 28.39, 23.85, 23.66, 21.89, 21.25, 19.58, 19.42, 15.27, 11.25, 11.20, 7.92, 7.89. MS (ESI): *m*/*z* 1048.2 ($M^+$ +1).

**Compound 5 -** TFA (5 mL) was added to a 0 °C solution of compound **4** (523 mg, 0.5 mmol) in DCM (100 mL). The resulting mixture was warmed to room temperature and stirred for 2 h, and evaporated under reduced pressure to give the crude product **5**. The crude product was used in the subsequent reaction without further purification. MS (ESI): *m*/*z* 948.6 ($M^+$ +1).

**Mal-azo-Exatecan -** Compound **5** was dissolved in dry DCM (50 mL) under nitrogen atmosphere, EMCS (847 mg, 3.5 mmol) and DIPEA (260 μL, 1.5 mmol) were added. The mixture was stirred at room temperature for 2 hours. The solvent was washed with 0.1 M hydrochloric acid (10 mL), brine (20 mL), dried over $Na_2SO_4$, filtered, and the solvent was evaporated under reduced pressure. The crude material was purified by column chromatography in 30:1 DCM/MeOH (v/v) to give **Mal-azo-Exatecan** (550 mg, yield 90%) as a light yellow solid. $^{1}$H NMR (400 MHz, CDCl3) δ 7.72 – 7.65 (m, 1H), 7.52 – 7.34 (m, 2H), 7.11 (d, J = 8.8 Hz, 1H), 6.65 (s, 2H), 6.58 – 6.30 (m, 1H), 6.18 (s, 1H), 5.59 – 5.51 (m, 1H), 5.30 – 5.19 (m, 2H), 5.13 – 4.88 (m, 2H), 4.67 – 4.38 (m, 6H), 4.25 – 3.96 (m, 5H), 3.82 – 3.71 (m, 2H), 3.59 – 3.54 (m, 8H), 3.51 – 3.45 (m, 4H), 3.40 – 3.34 (m, 2H), 3.19 – 3.05 (m, 2H), 2.35 (s, 3H), 2.21 (s, 2H), 2.13 – 2.08 (m, 2H), 1.97 – 1.88 (m, 2H), 1.60 – 1.50 (m, 4H), 1.31 – 1.22 (m, 2H), 1.07 – 1.00 (m, 6H), 0.91 (d, J = 7.4 Hz, 3H).$^{13}$C NMR (101 MHz, CDCl3) δ 173.51, 172.88, 170.85, 163.55, 163.52, 161.05, 161.02, 157.08, 156.99, 156.11, 156.00, 152.16, 152.14, 150.13, 150.07, 148.30, 145.82, 145.52, 145.40, 143.70, 143.53, 139.76, 139.43, 136.60, 136.49, 135.20, 135.15, 134.07, 129.19, 129.14, 125.18, 124.99, 124.79, 124.62, 124.50, 124.45, 121.27, 121.18, 119.50, 119.32,

113.92, 110.18, 110.03, 109.95, 109.78, 97.82, 75.89, 75.60, 72.82, 72.79, 70.71, 70.43, 70.41, 70.38, 70.36, 70.32, 70.12, 70.10, 69.87, 69.86, 69.23, 69.21, 66.03, 65.85, 62.76, 50.16, 49.56, 47.95, 47.84, 40.48, 40.27, 39.10, 37.62, 36.29, 35.29, 35.22, 31.41, 28.23, 26.33, 25.06, 21.85, 21.27, 19.60, 19.44, 15.27, 11.27, 11.22, 7.92, 7.90. HRMS（ESI）*m*/*z* calcd for $C_{55}H_{66}FN_{11}O_{15}Na$ [M + Na]$^+$: 1162.4622; found 1162.4543.

## 4.2. NMR raw spectra and high resolution mass spectra

**Mal-azo-Exatecan:**

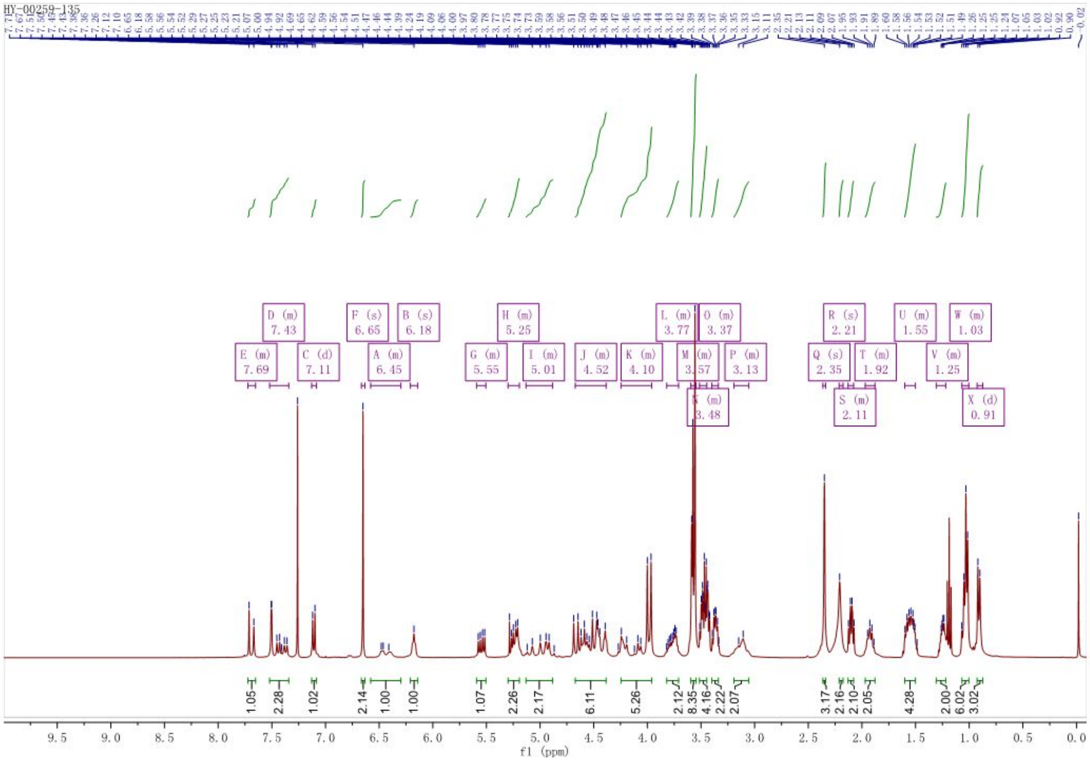

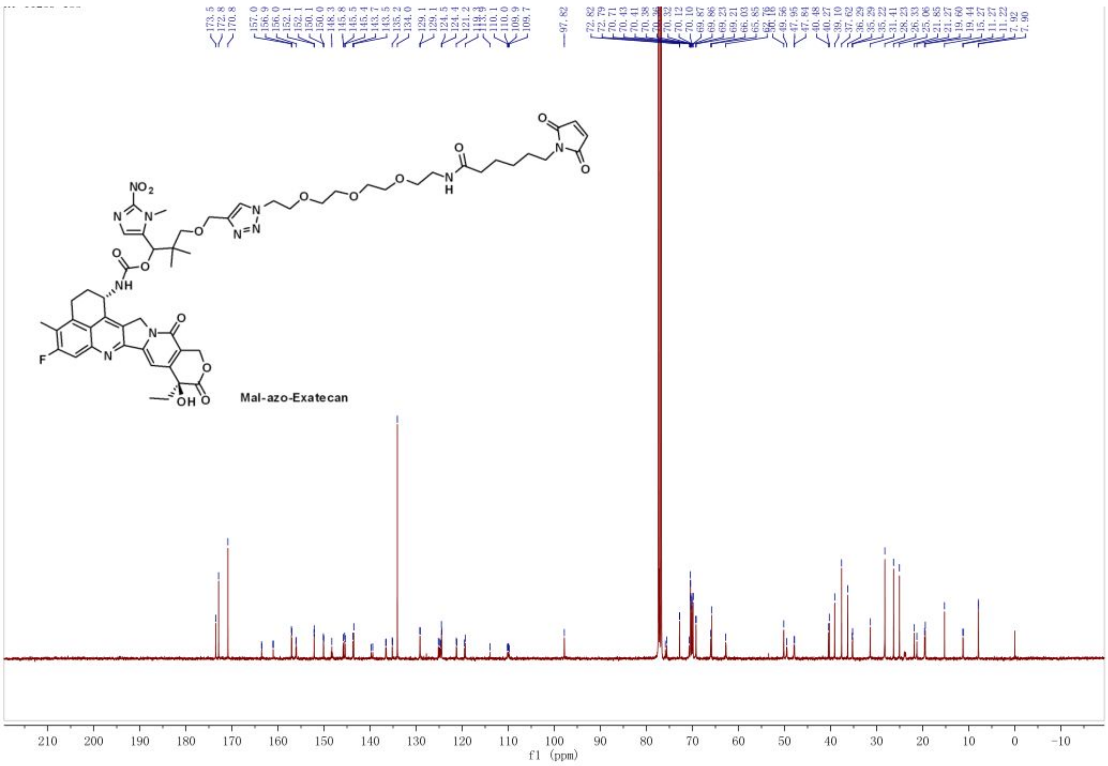


**Elemental Composition Report** **Page 1**

**Single Mass Analysis**
Tolerance = 500.0 mDa / DBE: min = -1.5, max = 50.0
Element prediction: Off

Monoisotopic Mass, Even Electron Ions
1 formula(e) evaluated with 1 results within limits (up to 50 closest results for each mass)
Elements Used:
C: 55-55 H: 52-69 N: 11-11 O: 15-15 Na: 0-1 F: 1-1

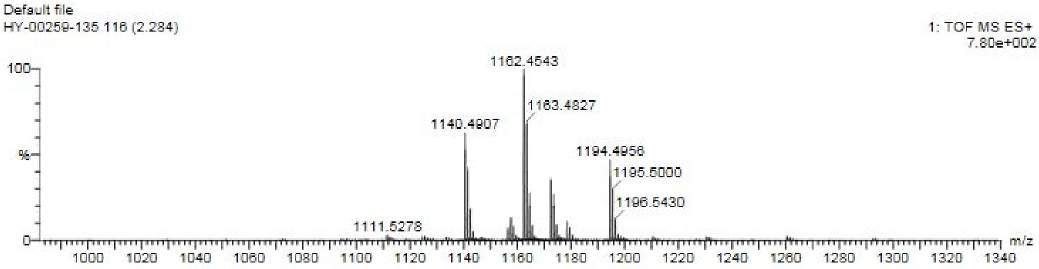


| | | | | | |
|---|---|---|---|---|---|
| Minimum: | | | | -1.5 | |
| Maximum: | | 500.0 | 10.0 | 50.0 | |
| Mass | Calc. Mass | mDa | PPM | DBE | Formula |
| 1162.4543 | 1162.4622 | -7.9 | -6.8 | 27.5 | C55 H66 N11 O15 Na F |
